# Antivirals Against Coronaviruses: Candidate Drugs for SARS-CoV-2 Treatment?

**DOI:** 10.3389/fmicb.2020.01818

**Published:** 2020-08-13

**Authors:** Igor de Andrade Santos, Victória Riquena Grosche, Fernando Rodrigues Goulart Bergamini, Robinson Sabino-Silva, Ana Carolina Gomes Jardim

**Affiliations:** ^1^Laboratory of Virology, Institute of Biomedical Science, Federal University of Uberlândia, Uberlândia, Brazil; ^2^Institute of Biosciences, Language and Exact Sciences, São Paulo State University, São José do Rio Preto, Brazil; ^3^Laboratory of Synthesis of Bioinspired Molecules, Institute do Chemistry, Federal University of Uberlândia, Uberlândia, Brazil; ^4^Department of Physiology, Institute of Biomedical Sciences, Federal University of Uberlândia, Uberlândia, Brazil

**Keywords:** antivirals, coronaviruses, COVID-19, SARS-CoV-2, treatment

## Abstract

Coronaviruses (CoVs) are a group of viruses from the family *Coronaviridae* that can infect humans and animals, causing mild to severe diseases. The ongoing pandemic of severe acute respiratory syndrome coronavirus 2 (SARS-CoV-2) represents a global threat, urging the development of new therapeutic strategies. Here we present a selection of relevant compounds that have been described from 2005 until now as having *in vitro* and/or *in vivo* antiviral activities against human and/or animal CoVs. We also present compounds that have reached clinical trials as well as further discussing the potentiality of other molecules for application in (re)emergent CoVs outbreaks. Finally, through rationalization of the data presented herein, we wish to encourage further research encompassing these compounds as potential SARS-CoV-2 drug candidates.

## Introduction

**FIGURE 1:**
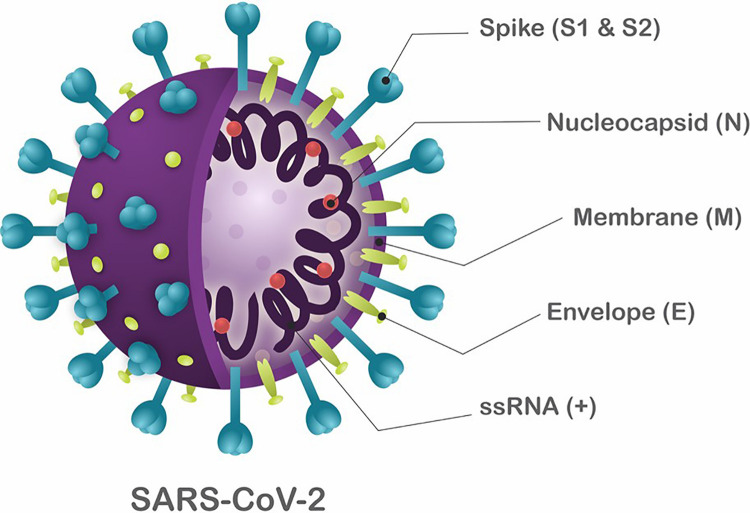
Schematic structure of SARS-CoV-2. The viral structure is primarily formed by the structural proteins such as spike (S), membrane (M), envelope (E), and nucleocapsid (N) proteins. The S, M, and E proteins are all embedded in the viral envelope, a lipid bilayer derived from the host cell membrane. The N protein interacts with the viral RNA in to the core of the virion.

Coronaviruses (CoVs) were first identified in 1960 (Kahn and McIntosh, 2005) and were classified as members of the family *Coronaviridae*. CoVs are enveloped, single-stranded RNA viruses with a genome varying from 25 to 32 kb (Payne, 2017). The viral structure is primarily formed by the structural spike (S), membrane (M), envelope (E), and nucleocapsid (N) proteins. The S, M, and E proteins are embedded in the viral envelope, which is a lipid bilayer derived from the host cell membrane. The N protein, on the other hand, interacts with the viral RNA into the core of the virion (Figure 1; Fehr and Perlman, 2015).

These viruses can infect vertebrate animals, causing acute to chronic diseases in the respiratory, cardiac, enteric, and central nervous systems, both in animals and humans (Weiss and Navas-Martin, 2005). In animals, the most common CoVs are infectious bronchitis virus (IBV), feline CoV (FeCoV), and mouse hepatitis virus (MHV), which infect chickens, felines, and rodents, respectively (Cui et al., 2019). To date, there are seven known CoVs that cause diseases in humans: HCoV-229E, HCoV-NL63, HCoV-OC43, HCoV-HKU1, severe acute respiratory syndrome coronavirus (SARS-CoV), Middle East respiratory syndrome coronavirus (MERS-CoV) and, most recently, SARS-CoV-2 (Graham et al., 2013; CDC, 2020a). The CoVs HCoV-229E, HCoV-NL63, HCoV-OC43, and HCoV-HKU1 cause mild symptoms, similar to a common cold (Payne, 2017). However, SARS-CoV, MERS-CoV, and SARS-CoV-2 can cause mild to severe symptoms related to upper respiratory infection such as fever, cough, dyspnea, pneumonia, and acute respiratory distress syndrome (ARDS), ultimately leading to death (Lai et al., 2020). The severe clinical condition generated especially by SARS-CoV-2 has been burdening public health systems worldwide (Hsu et al., 2020), evidencing the mandatory need for further research encompassing antiviral treatment against CoVs, which has somehow, until recently, been relatively ignored by broad pharmaceutical and medicinal fields (Lu et al., 2015; Cui et al., 2019).

CoVs are linked to a zoonotic transmission due to their ability to infect different species. This can lead to host jumps, allowing the emergence of new coronaviruses such as SARS-CoV, MERS-CoV, and SARS-CoV-2 (Lu et al., 2015; Reusken et al., 2016; Andersen et al., 2020). The transmission of CoVs is based on the fecal-oral route in animals (Kipar et al., 2010). In humans, CoV transmission occurs by direct contact with droplets when infected and recipient individuals are in close contact (about one meter). These infectious oral and respiratory droplets produced by talking, coughing, sneezing need to contact the mucosae (mouth and nose) or conjunctiva (eyes) of the recipient person. Additionally, indirect transmission can occur by touching a surface with viable CoV and subsequent contact with mouth, nose, or eyes (van Doremalen et al., 2020). Viral particles may remain viable on surfaces for several days, increasing the probability of infection by third parties (van Doremalen et al., 2020).

Recently, the emergence of SARS-CoV-2 was related to zoonotic transmission, but it is still not clear how this virus was first transmitted to humans (Andersen et al., 2020; Gorbalenya et al., 2020b). By phylogenetic analysis, the SARS-CoV-2 was grouped within bat SARS-related coronaviruses, suggesting that a host jump occurred (Cao et al., 2020a; Lai et al., 2020). Alarmingly, the high transmissibility of this new CoV allowed the rapid and efficient spread of the virus across the world so that it became a pandemic disease in just a few months (CDC, 2020a; Wu et al., 2020).

**FIGURE 2:**
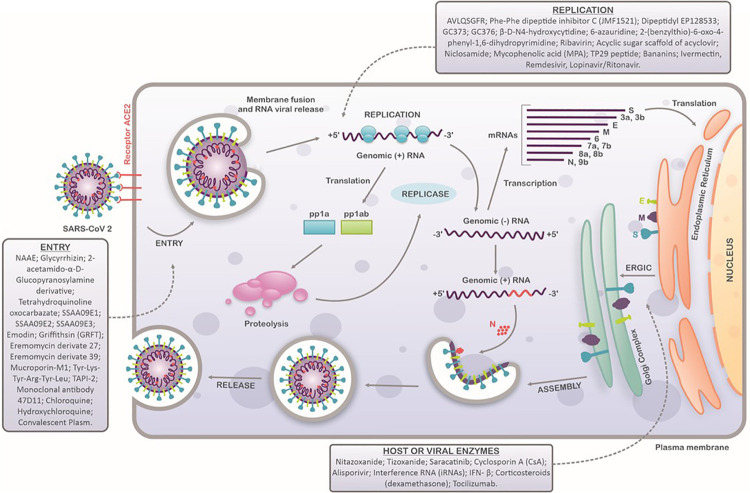
Schematic representation of SARS-CoV-2 replication cycle in host cells. SARS-CoV-2 attaches to the host cells by interaction between the ACE2 receptors and spike proteins. After entry, viral uncoating process results in the release of viral genome and replication stage occurs (translation and transcription). Structural proteins are produced in intermediate compartment of endoplasmic reticulum with Golgi complex and forwarded to assembly, packaging and virus release. Compounds with antiviral activity against SARS-CoV-2 are indicated in each step of virus replication cycle.

Due to the novelty of this disease, there is a lack of understanding of the SARS-CoV-2 replication process in host cells. The general mechanisms of entry into the host cell, replication, and release follow characteristics that have been described for other CoVs and have been partially confirmed for SARS-CoV-2. To date, it is known that the SARS-CoV-2 virion entries the host cells by the attachment of the S protein with angiotensin-converting enzyme 2 receptor (ACE2), defining SARS-CoV-2 tropism for cells that express this receptor, such as pulmonary, hepatic, gastrointestinal, and renal human cells (Chu et al., 2020; Hoffmann et al., 2020; Tai et al., 2020). The interaction of ACE2 with the receptor-binding domain (RBD) of the S protein triggers virion endocytosis and the formation of an endosome (Rabi et al., 2020). The S protein possesses two subunits, S1 and S2 (Walls et al., 2020). During endocytosis, an acid-dependent proteolytic cleavage of the S1 protein by cellular proteases, like cathepsin, TMPRRS2, and trypsin, exposes the S2 subunit, a fusion peptide that allows the fusion of the viral envelope with the endosome membrane, and consequently, releases the capsid into the cell cytoplasm (Belouzard et al., 2009; Matsuyama et al., 2020). In the cytoplasm, the CoV viral genome is uncoated, and the viral RNA is released. The positive-sense RNA viral genome is translated to produce nonstructural proteins (nsps) from two open reading frames (ORFs), ORF1a and ORF1b. The ORF1a encodes the polyprotein pp1a that is cleaved in 11 nsps, while the ORF1b encodes the polyprotein pp1ab, which is cleaved into 15 nsps. The proteolytic cleavage is performed by viral proteases nsp3 and nsp5 (Yogo et al., 1977; Lai and Stohlman, 1981; Kim et al., 2020). The nsps assemble to form a replicase-transcriptase complex (RTC) responsible for RNA synthesis, replication, and transcription of nine subgenomic RNAs (sgRNAs) (Fehr and Perlman, 2015; Chen W.-H. et al., 2020; Kim et al., 2020). The sgRNAs act as mRNAs for structural and accessory genes localized downstream of the replicase polyproteins. SARS-CoV-2 has six accessory proteins: 3a, 6, 7a, 7b, 8, and 10 (Kim et al., 2020). The structural proteins S, E, and M are translated from the sgRNAs and forwarded to the endoplasmic reticulum (ER) and are subsequently inserted into an intermediate compartment of ER with Golgi (ERGIC). There, viral genomes are encapsulated by N proteins and assembled with the structural proteins to form virions (Siu et al., 2008; Fehr and Perlman, 2015; Li et al., 2020). The M proteins bind to E protein and nucleocapsid, and then, the S protein is incorporated, forming a complete virion. Finally, the virions are transported to the cell surface in vesicles and released in a pathway mediated by exocytosis (Figure 2; Fehr and Perlman, 2015; Kim et al., 2020; Li et al., 2020).

It is important to emphasize that SARS-CoV-2 shows different epidemiological and clinical features from the epidemics of SARS-CoV and MERS-CoV (Ceccarelli et al., 2020; Gorbalenya et al., 2020a, b). The high transmissibility of SARS-CoV-2 may be related to its entry into host cells (Sun et al., 2020). Although both SARS-CoV and SARS-CoV-2 glycoprotein S attach to ACE2 to enter the host cells, the binding affinity of SARS-CoV-2 is higher, thus enhancing its infectivity (Sun et al., 2020; Yan et al., 2020). Despite the relative homology between S1 and S2 amino acid sequences, a 1.2 Å root-mean-square deviation at the 417 position (Lusvarghi and Bewley, 2016) of S2 protein in SARS-CoV-2 may be related to its higher infectiveness, contributing to a 10- to 20-fold higher kinetic affinity of SARS-CoV-2 ectodomain, as evidenced by Wrapp and co-workers, employing surface plasmon resonance measurements (Wrapp et al., 2020).

Considering the particularities of SARS-CoV-2 and the emergency caused by its outbreak, several strategies have been adopted to develop therapeutics and prophylactic measures against this virus. The strategies employed in these developments include: (i) utilization of bioinformatics for the prediction and investigation of potential ligands toward target molecules in the viral structure and/or replication (Ahmed et al., 2020, 2; Jeon et al., 2020, 2); (ii) employment of cell culture systems, permissive to CoVs (Caly et al., 2020; Liu et al., 2020), associated with pseudo particles, subgenomic replicons and/or full-length CoVs, seeking to assess cellular response or the effects of the compounds on the viral replicative cycle (Roberts et al., 2006; Hoffmann et al., 2020); (iii) the use of animal models, such as mice, mouse, guinea pig, hamster and non-human primates, for evaluating therapeutic options or antibody production in immunization (Natoli et al., 2020; Sheahan et al., 2020b), and (iv) clinical trials assessing the administration, distribution, metabolism, and toxicity profiles (ADMeTox) of potential therapeutics as well as immunization effects in humans (Clark et al., 2019).

Based in previous results in vaccine development for MERS-CoV and SARS-CoV and the similarity of those viruses with SARS-CoV-2 (Dhama et al., 2020), the current vaccine candidates are more focused on the S protein, since is a major inducer of neutralizing antibodies in infected patients (Walls et al., 2020). For this reason, efforts are concentrated on using approaches such as mRNA, DNA, viral vectors, or virus-like particles vaccines with a full-length S protein or S1 receptor-binding domain (RBD) to stimulate immune response and immunization (Ahmed et al., 2020; Chen Y. et al., 2020). The most promising vaccines are: (i) adenovirus-vectored AZD1222 produced by Oxford University (Thomas, 2020), a vaccine that is currently in clinical phase 3, being tested in several countries, including the United States, Brazil, and countries in Asia and Africa; (ii) mRNA-1273 associated with a lipidic nanoparticle (NCT04283461), which is currently in clinical phase 2; and (iii) inactivated virus vaccine, which is currently in clinical phase 1 (Mullard, 2020; Tu et al., 2020).

The high transmissibility and viral variability of the novel SARS-CoV-2, along with the lack of a vaccine or drugs to treat the infected patients, threaten the global health system. In this context, the development of effective antivirals is critical to provide short-term therapies able to reduce the severity of clinical outcomes of coronavirus disease 2019 (COVID-19) and to reduce the spread of SARS-CoV-2. Here, we summarize compounds described, from 2005 to date, to possess antiviral activity *in vitro* and/or *in vivo* against CoVs and critically compare molecules that could be further investigated by their clinical applicability (Table 1). We also discuss the compounds that have reached clinical trials (Table 2) as well as the potentiality of other molecules for application in (re)emergent CoVs outbreaks. Finally, we aim to encourage further research encompassing these compounds as potential SARS-CoV-2 drug candidates.

**TABLE 1:** Compounds with antiviral activity against human and animal coronaviruses.

Compound	Inhibition step	EC50 or inhibition (%)	CoVs	Advantges and/or limitations	References
NAAE	Entry	0.5 μM	SARS-CoV	Synthetic molecule, evaluated *in silico*, easily produced but lacks *in vivo* assays	Huentelman et al., 2004b
Glycyrrhizin	Entry	300 mg L^–1^	SARS-CoV	Natural molecule, highly tolerated but lacks *in vivo* assays	Cinatl et al., 2003
2-acetamido-α-D-Glucopyranosylamine derivative	Entry	40 μM	SARS-CoV	Semi-synthetic molecule, highly tolerated, and more potent inhibitor but lacks *in vivo* assays	Hoever et al., 2005
Tetrahydroquinoline oxocarbazate (CID 23631927)	Entry (Cathepsin L)	273 nM	SARS-CoV	Synthetic molecule, highly tolerated, easily produced but lacks *in vivo* assays	Shah et al., 2010
SSAA09E1	Entry	6.7 μM	SARS-CoV	Synthetic molecule, highly tolerated, easily produced but lacks *in vivo* assays	Adedeji et al., 2013
SSAA09E2	Entry	3.1 μM	SARS-CoV	Synthetic molecule, highly tolerated, easily produced but lacks *in vivo* assays	Adedeji et al., 2013
SSAA09E3	Entry	9.7 μM	SARS-CoV	Synthetic molecule, highly tolerated, easily produced but lacks *in vivo* assays	Adedeji et al., 2013
Emodin	Entry and Post-Entry	50 μM	SARS-CoV	Natural molecule, highly tolerated but lacks *in vivo* assays	Ho et al., 2007; Schwarz et al., 2011
Griffithsin (GRFT)	Entry	0.16 μg mL^–1^	HCoV-OC43	Natural molecule, highly tolerated, with a broad-spectrum effect (human and animal CoVs); protected against infection and improved survival in animal assay (Balb/c)	O’Keefe et al., 2010
	Entry	0.18 μg mL^–1^	HCoV-229E		
	Entry	0.61 μg mL^–1^	SARS-CoV		
	Entry	<0.032 μg mL^–1^	HCoV-NL63		
	Entry	0.057 μg mL^–1^	BCoV		
	Entry	0.23 μg mL^–1^	MHV		
Eremomycin derivate 27	Entry	5.4 μM	FIPV	The precursor molecule (Eremomycin) is used to treat bacterial infections; may facilitate clinical assays, but knowledge of the mechanism of action is lacking	Balzarini et al., 2006
	Entry	14 μM	SARS-CoV		
Eremomycin derivate 39	Entry	12 μM	FIPV		
	Entry	22 μM	SARS-CoV		
Mucroporin-M1	Entry	14.46 μg mL^–1^	SARS-CoV	Synthetic molecule, moderately tolerated, easily produced but lacks *in vivo* assays	Li et al., 2011
Tyr-Lys-Tyr-Arg-Tyr-Leu	Entry	14 mM	SARS-CoV	Synthetic molecule specifically designed to bind S protein of SARS-CoV; highly tolerated, does not impair ACE2 activity but lacks *in vivo* assays	Struck et al., 2012
	Entry	14 mM	HCoV-NL63		
TAPI-2	Entry	65%	SARS-CoV	Good effects in vitro assays but had no effect on *in vivo* assays	Haga et al., 2010
Monoclonal antibody 47D11	Entry	0.57 μg mL^–1^	SARS-CoV-2	Human antibody, specifically to SARS-CoV-2, highly tolerated and easily applicable	Wang et al., 2020a
AVLQSGFR	Replication	2.7 × 10^–2^ mg mL^–1^	SARS-CoV	Synthetic molecule, highly tolerated, easily produced but lacks *in vivo* assays	Gan et al., 2006
Phe-Phe dipeptide inhibitor C (JMF1521)	Replication	0.18 μM	SARS-CoV	Synthetic molecule, highly tolerated, easily produced but lacks *in vivo* assays	Shie et al., 2005
Dipeptidyl EP128533	Replication	3.6 μM or 1.4 μg mL^–1^	SARS-CoV	Synthetic molecule, highly tolerated, easily produced but has contrasting effects in the literature and did not inhibit the virus in *in vivo* assays	Zhang et al., 2006; Day et al., 2009
GC373	Replication	0.2 μM	HCoV-229E	Synthetic molecule, highly tolerated, easily produced, seems to interact with SARS-CoV 3CLpro, but there are no *in vivo* assays	Kim et al., 2012, 2013
		0.3 μM	FIPV		
		2 μM	MHV		
		0.3 μM	TGEV		
		0.7 μM	BCV		
		0.15 μM	FCoV-WSU		
GC376	Replication	0.15 μM	HCoV-229E	Synthetic molecule, highly tolerated, easily produced, seems to interact with SARS-CoV 3CLpro, but there are no *in vivo* assays	Kim et al., 2012, 2013
		0.2 μM	FIPV		
		1.1 μM	MHV		
		0.15 μM	TGEV		
		0.6 μM	BCV		
		0.40 μM	FCoV-WSU		
6-azauridine	Replication	32 nM	HCoV-NL63	Synthetic molecule, highly tolerated, easily produced, but there are no *in vivo* assays	Pyrc et al., 2006
2-(benzylthio)-6-oxo-4-phenyl-1,6-dihydropyrimidine	Replication	NE	SARS-CoV	Synthetic molecule, highly tolerated, easily produced, but there are no in vivo assays	Ramajayam et al., 2010
β-D-N^4^-hydroxycytidine	Replication	10 μM	SARS-CoV	Synthetic molecule, highly tolerated, easily produced, and improved pulmonary function and decreased viral load in lung of infected mice	Barnard et al., 2004; Sheahan et al., 2020b
	Replication	400 nM	HCoV-NL63		
	Replication	0.08–0.3 μM	SARS-CoV-2		
	Replication	0.024 μM	MERS-CoV		
Ribavirin	Replication	20 μg mL^–1^	SARS-CoV	Synthetic molecule, highly tolerated, easily produced, good results in MERS-CoV. However, meta-analyses indicate limited efficacy.	Saijo et al., 2005; Barnard et al., 2006
Acyclic sugar scaffold of acyclovir	Replication	23 μM	MERS-CoV	Synthetic molecule, highly tolerated, easily produced, but there are no *in vivo* assays	Peters et al., 2015
		8.8 μM	HCoV-NL63	Synthetic molecule, highly tolerated, derivate from Acyclovir, easily produced, but there are no in vivo assays	
Niclosamide	Replication	0.1 μM	SARS-CoV	Drug already in use to treat helminthic infections; good inhibition *in vitro*	Wu et al., 2004; Wen et al., 2007
Mycophenolic acid (MPA)	Replication	2.87 μM	MERS-CoV	Good effects *in vitro* with MERS-CoV but did not inhibit SARS-CoV in *in vitro* and *in vivo* assays	Cinatl et al., 2003; Barnard et al., 2006; Hart et al., 2014
TP29 peptide	Replication	60 μM	MHV	Inhibited two species of CoV in mice; also improved survival and induced INF-I. Inhibited CoV in cell lines. Synthetic compound designed for nonstructural proteins.	Wang et al., 2015
	Replication	200 μM	SARS-CoV		
Bananins	Replication	<10 μM	SARS-CoV	Synthetic molecule, highly tolerated, easily produced, but there are no *in vivo* assays	Tanner et al., 2005
Nitazoxanide	Host Enzymes	0.92 μg mL^–1^	MERS-CoV	Drug already in use to treat viral infections; good inhibition *in vitro*	Rossignol, 2016
Tizoxanide	Host Enzymes	0.83 μg mL^–1^	MERS-CoV	Drug derived from Nitazoxanide; good inhibition *in vitro*	Rossignol, 2016
Saracatinib	Tyrosine Kinases	2.9 μM	MERS-CoV	Synthetic molecule, highly tolerated, used to treat Alzheimer’s disease and easily produced but there are no in vivo assays	Shin et al., 2018
Cyclosporin A (CsA)	Hosts Cyclophilin Family Enzymes	9–32 μM	SARS-CoV, MERS-CoV and MHV	Drug already used to treat several chronic and infectious diseases with broad-spectrum activity among CoVs	de Wilde et al., 2011, 2013; Pfefferle et al., 2011
Alisporivir	Hosts Cyclophilin Family Enzymes	8.3 μM	SARS-CoV	Analog of CsA and has a strong inhibition *in vitro* against SARS-CoV and other CoVs	de Wilde et al., 2017
Interference RNA (iRNAs)	Viral Proteins Translation	70%	SARS-CoV	Different approach, specific targeting of viral proteins; can block replication steps and has no cytotoxicity	Åkerström et al., 2007
	Viral Proteins Translation	99%	SECoV	Different approach, specific targeting of viral proteins; can block replication steps and has no cytotoxicity	Li et al., 2019

*EC50: effective concentration of 50%; NE: not evaluated.*

**TABLE 2:** Ongoing clinical trials of candidate drugs against SARS-CoV-2 in COVID-19 patients.

Drug	Cell culture assays	Inhibition step *in vitro*	Animal assays	Clinical trials	Outcomes in clinical trials	Advantages and/or limitations
Remdesivir	Inhibited SARS-CoV, MERS-CoV, and SARS-CoV-2	Replication (RdRp)	Inhibited EBOV and SARS-CoV in both infected mice and monkeys	Clinical case and clinical trial against SARS-CoV-2	Did not provide antiviral effects or improved clinical outcomes	This is a multicentre, double-blind, placebo-controlled clinical trial, but more studies might be needed to confirm, since this includes 255 people, and the drug has some adverse effects.
Lopinavir and Ritonavir	Inhibited SARS-CoV and MERS-CoV	Replication (protease inhibitor)	NE	Clinical trial with SARS-CoV-2	Did not provide antiviral effects or improved clinical outcomes in severe patients, but, in early infections, clinical outcomes were improved.	This drug combination is used for other human CoVs, but the study was not multicentre, double-blind, and placebo-controlled. More studies are necessary to confirm, since it had only 199 people and the drug showed some adverse effects.
IFN-β	Inhibited SARS-CoV, MERS-CoV, MHV, and HCoV-229E	Host Factors (inducing immune response)	Inhibited SARS-CoV, MERS-CoV, MHV, and HCoV-229E	Clinical trial with SARS-CoV-2 and is used for other diseases	Do not have effect alone	IFN-β is indicated to be safe, with few adverse effects, but in clinical trials, it is only effective when associated with other drugs.
Umifenovir	Inhibited SARS-CoV	NE	NE	Observational study with 81 patients	Did not provide antiviral effects or improved clinical outcomes	This is an observational study and might suffer bias from lack and/or loss of information and data. It is an applicable study, since it demonstrates a tendency, and the drug is already used to treat Influenza viruses.
Corsticosteroids (dexamethasone)	NE	Host factors (controlling immune response)	NE	Clinical trial with 454 treated patients	Reduced death by one-third in invasive mechanical ventilation patients and one-fifth in oxygen without invasive mechanical ventilation patients; however, did not impair mortality in patients without respiratory support	This is a multicentre, double-blind, placebo-controlled clinical trial. More studies are needed to understand better the effect on different phases of COVID-19. May be a good alternative for treating hyperinflammation and hypersecretion of cytokines.
Ivermectin	Inhibited SARS-CoV-2 and arboviruses (CHIKV and DENV)	Replication (nonstructural proteins)	NE	Clinical trials are beginning	NE	Ivermectin is safe for use in humans since it is used to treat several parasitic infections.
Tocilizumab	NE	Inhibitor of IL-6	NE	Ongoing clinical trials with SARS-CoV-2 patients; one with 100 patients concluded.	Positive effects: improved inflammatory markers and decreased the need for ventilatory support in patients	Tocilizumab is already used to treat viral infections, controlling immune response, impairing cytokine storms, improving antiviral response, and providing the best clinical outcomes.
Chloroquine	Inhibited HIV, CHIKV, SARS-CoV, and SARS-CoV-2	Entry	Improved outcomes in FCoV positive cats	Several clinical trials are being conducted	Impairs virus replication and has anti-inflammatory activities	Chloroquine possesses important side effects and is indicated only in severe cases. However, there are some studies with contrasting results regarding its safety, since it can cause arrhythmias, hypoglycemia, neuropsychiatric effects, and depression.
Hydroxychlo-roquine	Inhibited HIV, CHIKV, SARS-CoV, and SARS-CoV-2	Entry	NE	Several clinical trials are being conducted	Less toxic option, impairs virus replication	Hydroxychloroquine improved patients’ outcomes, including when associated with azithromycin. Less toxic option than chloroquine treatment, but there are studies with contrasting results regarding its safety, since it can cause arrhythmias, hypoglycemia, neuropsychiatric effects, and depression.
Convalescent Plasma	NE	Entry	NE	Case report	Improved outcomes and suppressed viremia.	Administration poses a risk to patients since it is related to donor-dependent variability and compatibility. Antibody titers may interfere with its activity. In addition, it might cause side effects in lung and the cardiovascular system.

*EC50: effective concentration of 50%; NE: not evaluated.*

## Inhibitors of the CoV Replicative Cycle

### Inhibitors of CoV Entry Into Host Cells

The entry of human CoVs into the host cells is mainly related to the binding of viral S protein to the ACE2 receptor (Prabakaran et al., 2004; Sun et al., 2020). Therefore, it is reasonable to hypothesize that compounds affecting this interaction could be potential antivirals (Prabakaran et al., 2004).

In this context, a survey encompassing *in silico* studies of more than 140 thousand potential S-protein-inhibiting drugs indicated that the molecule *N*-(2-aminoethyl)-1 aziridineethanamine (NAAE) showed the highest docking grade (-23.7 kcal/mol) (Huentelman et al., 2004b). The activity of NAAE was further confirmed by employing an *in vitro* enzymatic inhibitory assay, using a human recombinant ACE2. In this assay, ACE2 removed the C-terminal dinitrophenyl moiety that quenched the inherent fluorescence of the 7-methoxycoumain group, increasing the fluorescence when ACE2 was active (Huentelman et al., 2004b). The results showed that NAAE inhibited the ACE2 enzymatic activity with the half maximal inhibitory concentration (IC_50_) of 57 μmol mL^−1^ (Huentelman et al., 2004b). In addition, 293T cells expressing ACE2 receptor were incubated with NAAE and then with S glycoprotein-expressing 293T cells, and measurement of β-galactosidase activity (reported gene in cell-cell fusion) was performed. NAAE at 0.5 μM inhibited 50% of SARS-CoVs spike protein-mediated cell fusion, suggesting that NAAE might be a candidate for treating SARS infection by impairing viral attachment via interference with ACE2 (Huentelman et al., 2004b). However, a detailed explanation of how NAAE is a more efficient ligand to ACE2 than other compounds was not attempted by the authors.

Ramos-Tovar and Muriel reported the antiviral activity of Glycyrrhizin (GL), a major constituent from licorice root (Ramos-Tovar and Muriel, 2019), which was able to inhibit SARS-CoV entry into Vero cells with an effective concentration of 50% (EC_50_) of 300 mg L^–1^ and a cytotoxicity concentration of 50% (CC_50_) of >20.000 mg L^–1^. GL was less effective when the administration occurred during the viral adsorption period than when it was administered after entry into host cells. Cumulative effects were observed when this compound was administered both during and after entry into host cells, which indicates a significantly potent inhibitor against the virus under the tested conditions (Cinatl et al., 2003). Additionally, the antiviral activity of 15 GL derivates against SARS-CoV was assessed (Hoever et al., 2005). Conjugation on both acidic moieties of the GL disaccharide group with 2-acetamido-α-D-glucopyranosylamine, benzylcysteine, and Gly-Leu peptide generated compounds with an increase of 10- to 70-fold in anti-SARS-CoV activity when compared to GL itself (Hoever et al., 2005). For the case of 2-acetamido-α-D-glucopyranosylamine derivative, it was speculated that viral entry was inhibited through *N*-acetylglycosamine binding onto S-protein carbohydrates. Other derivatives such as the introduction of heterocyclic amides such as 6-amine-thiouracil induced a higher cytotoxicity profile.

The endossomal cathepsins are essential enzymes in viral entry into host cells (Huang et al., 2006), and cathepsin L has been pointed to as playing a crucial role in membrane fusion with the endosomes (Belouzard et al., 2009; Matsuyama et al., 2020). In this context, Shah and coworkers demonstrated the effective activity of tetrahydroquinoline oxocarbazate (CID 23631927), an oxocarbazate inhibitor of cathepsin L, against SARS-CoV. Employing a pseudovirus system with a luciferase reporter to infect 293T cells, the compound inhibited viral entry with an EC_50_ of 273 ηM and CC_50_ > 100 μM (Shah et al., 2010). The authors also showed that the compound CID 23631927 seems to bind with a lower inhibition constant (K_*i*_) to cathepsin L, improving the compound/cathepsin L interaction. This might be related to its optimized structure, with stronger hydrophobic interactions and better hydrogen bonds between the compound and cathepsin L (Shah et al., 2010).

An extensive study screened a library of compounds following Lipinski’s rule (Lipinski et al., 2001) and identified three noncytotoxic compounds capable of inhibiting SARS-CoV pseudoparticle entry into 293T cells (Adedeji et al., 2013). *N*-(9,10-dioxo-9,10-dihydroanthracen-2-yl)benzamide (SSAA09E1) blocked early interactions of SARS-CoV S protein with ACE2 (EC_50_ of 6.7 μM and CC_50_ > 100 μM), whereas *N*-[[4-(4-methylpiperazin-1-yl)phenyl]methyl]-1,2-oxazole-5-carboxamide (SSAA09E2) affected cathepsin L activity (EC_50_ of 3.1 μM and CC_50_ > 100 μM). Conversely, [(Z)-1-thiophen-2-ylethylideneamino]thiourea (SSAA09E3) prevented the fusion of the viral envelope with host membrane cells by direct interaction with spike protein (EC_50_ of 9.7 μM and CC_50_ > 20 μM) (Adedeji et al., 2013). The compound SSAA09E3 presented the highest cytotoxic, probably due to the interactions with host proteins. The authors suggested that since these three compounds are derived from molecules with antiviral activities and presented good oral bioavailability and rapid systemic distribution in animal models, they might exhibit interesting pharmacokinetics (Adedeji et al., 2013).

Other compounds also demonstrated to inhibit CoV entry, for example, emodin (6-methyl-1,3,8-trihydroxyanthraquinone), a component from *Rheum officinale* roots, which at 50 μM inhibited the infectivity of S protein-pseudotype retrovirus from SARS-CoV in Vero cells by about 80% (Ho et al., 2007). Besides the entry activity, emodin was described to have an additional post-entry antiviral action. The authors suggested that emodin might be impairing virus release by affecting 3a viral protein, which is related to ion channels in infected Vero cells (Schwarz et al., 2011). This effect may play an important role in immune response.

The exploitation of other natural compounds such as proteins as potential anti-CoV drugs has also been performed. Griffithsin (GRFT) is a protein isolated from the red alga *Griffithsia* sp. that has shown powerful viral entry inhibition against several enveloped viruses, such as the human immunodeficiency virus (HIV). GRFT is capable of binding to terminal mannoses of oligosaccharides and also to glycans localized on the viral envelope glycoproteins (Lusvarghi and Bewley, 2016). GFRT did not present cytotoxicity in Vero cells, human ileocecal colorectal adenocarcinoma cells, human diploid fibroblast cells, and rhesus monkey kidney cells. Its broad-spectrum antiviral activity *in vitro* was demonstrated against several CoVs such as SARS-CoV (EC_50_ of 0.61 μg mL^−1^), bovine coronavirus (BCoV) (EC_50_ of 0.057 μg mL^–1^), MHV (EC_50_ of 0.23 μg mL^–1^), HCoV-OC43 (EC_50_ of 0.16 μg mL^–1^), HCoV-229E (EC_50_ of 0.18 μg mL^–1^), and HCoV-NL63 (EC_50_ < 0.032 μg mL^–1^) (O’Keefe et al., 2010). In another study, GRFT inhibited the early stages of MERS-CoV infection in HEK-293T cells (Millet et al., 2016). Furthermore, GRFT improved survival in SARS-CoV-infected mice and protected the Balb/c female mice against infection by binding with S protein (O’Keefe et al., 2010). Altogether, this evidence indicates that GRFT can be considered as a potential SARS-CoV-2 entry inhibitor with activity against S proteins.

Antiviral activity by entry inhibition was also evaluated by employing antibacterial chemotherapeutics. Vancomycin, eremomycin, and teicoplanin glycopeptide compounds used to treat infections caused by Gram-positive bacteria (Preobrazhenskaya and Olsufyeva, 2004), as well as hydrophobic derivatives of these drugs, were described to possess antiviral activity against HIV (Printsevskaya et al., 2005). A study showed that vancomycin, eremomycin, and teicoplanin were not toxic to Vero and T lymphoblast (CEM) cells. Nonetheless, these compounds were not able to inhibit feline CoV (FIPV) and SARS-CoV in assays employing such cell lines. Conversely, the eremomycin derivative molecules labeled 27 and 39 showed the best inhibition profiles against FIPV (EC_50_ of 5.4 and 12 μM, respectively) and SARS-CoV (EC_50_ of 14 and 22 μM, respectively) (Balzarini et al., 2006).

Cationic antimicrobial peptides (AMPs) are another type of peptides that have been considered as potential broad-spectrum antiviral agents. For instance, mucroporin is an AMP found in *Lychas mucronatus* scorpion venom (Dai et al., 2008). Mucroporin was then optimized synthetically, generating mucroporin-M1, which was able to inhibit measles virus (MeV), SARS-CoV, and influenza H5N1. Specifically, mucroporin M-1 affected SARS-CoV pseudovirus entry, with EC_50_ of 14.46 μg mL^–1^ and CC_50_ of 61.58 μg mL^–1^, by virucidal activity in HeLa-ACE2 cells (Li et al., 2011). The activity of this synthetic peptide seems to be related to positive charges of the hydrophilic site, which can enhance the interaction with the viral surface, inactivating the viral particle.

Other potential antiviral peptides were selected by Struck and colleges. Through the exploitation of bioinformatics tools, the authors were able to predict sixteen peptides with effective binding onto the receptor-binding domain (RDB) present in S proteins of CoVs. These compounds were then synthesized, and the hexapeptide Tyr-Lys-Tyr-Arg-Tyr-Leu at 14 mM inhibited SARS-CoV and HCoV-NL63 infection in Vero cells without triggering cytotoxicity (Struck et al., 2012). This peptide was designed specifically to bind to the site of interaction with S protein and does not interfere with ACE2 receptor activity, so it might be a good candidate for blocking SARS-CoV-2 entry without impairing host metabolism. Taking into consideration that cellular factors such as the Tumor Necrosis Factor-alpha (TNF-α) converting enzyme (TACE) facilitate SARS-CoV entry (Haga et al., 2008), it is reasonable to suggest that TACE inhibitors could hinder SARS-CoV infection. In this context, TAPI-2, a compound able to inhibit TACE, has shown potent antiviral activity, promoting a 65% blockade of SARS-CoV entry in HEK-293T cells. However, the compound did not affect the virus titer in *in vivo* assays (Haga et al., 2010). The authors suggested that since SARS-CoV attaches to additional receptors such as DC-SIGN and L-SIGN (Jeffers et al., 2004; Han et al., 2007), viral entry might be not be impaired by this molecule.

In addition to amino acid-based inhibitors, monoclonal antibodies (mAbs) have attracted attention due to their use in infectious and chronic disease treatments (Green et al., 2000; Haynes et al., 2009; Pettitt et al., 2013; D’Amato et al., 2014), overcoming drawbacks caused in polyclonal Abs therapy, such as those related to donor compatibility (Marasco and Sui, 2007). Human neutralizing Abs against human CoVs have been generated, targeting S glycoproteins to impair viral entry (Belouzard et al., 2012; Reguera et al., 2012). Notably, several mAbs were identified as inhibitors of MERS-CoV and SARS-CoV infections both *in vitro* and *in vivo*, protecting cells and animals when administered 24 h prior to or post-infection (Lip et al., 2006; Zhu et al., 2007; Agnihothram et al., 2014; Shanmugaraj et al., 2020). The mAbs are developed by merging B lymphocytes and myeloma cells, producing hybridomas capable of recognizing antigens and producing a single Ab class to bind specific epitopes (Lipman et al., 2005). For that reason, mAb cross-reactivity among different coronaviruses seems to be ineffective (Totura and Bavari, 2019). In the particular case of SARS-CoV-2, Wang and coworkers produced mAbs using 51 lineages of SARS-S hybridoma cells and identified 47D11 H2L2-neutralizing Ab through ELISA assays. This antibody was produced using mice cells; therefore, it was further modified to produce a fully human immunoglobulin IgG1, producing the human monoclonal antibody 47D11. The results showed that 47D11 bound to the RBD region and inhibited SARS-CoV-2 entry in Vero cells with an EC_50_ of 0.57 μg mL^−1^ (Wang et al., 2020a). In this context, this mAb can be used alone or in association with other compounds to treat COVID-19.

### Inhibitors of Post-entry Stages of the CoV Replicative Cycle

Among the proteins that are pivotal for CoV viral replication are the main proteases (Mpro) such as the chymotrypsin-like protease (3CLpro) and the papain-like proteases (PPL). These enzymes process viral polyproteins and control replicase complex activity (Anand et al., 2003), figuring as very attractive targets for drug development against CoVs. Several natural products and synthetic peptides have been reported to inhibit Mpro (Cinatl et al., 2005; Vuong et al., 2020).

Gan and coworkers used molecular docking methods to select the octapeptide Ala-Val-Leu-Gln-Ser-Gly-Phe-Arg as Mpro inhibitor of SARS-CoV and evaluated its antiviral activity in infected Vero cells. The octapeptide presented an EC_50_ of 2.7 × 10^–2^ mg mL^–1^ and a CC_50_ > 100 mg mL^–1^, resulting in a selectivity index of over 3,704 (Gan et al., 2006). Moreover, five Phe-Phe dipeptide inhibitors (A-E) were designed and selected *in silico* to interact with 3CLpro and showed to be able to protect Vero cells from the cytopathic effect (CPE) caused by SARS-CoV. C analog (JMF1521) was obtained by the condensation of Phe-Phe dipeptide unsaturated ester with cinnamic acid and exhibited the highest activity, with an EC_50_ of 0.18 μM and CC_50_ > 200 μM (Shie et al., 2005). The authors also performed enzymatic assay to evaluate the activity of JMF1521 on 3CLpro and showed that the peptide inhibited the 3CLpro activity with an inhibition constant of 0.52 μM. The results suggested that this analog disposes a rather rigid coplanar structure in the N-terminal motif that results in more effective hydrogen bonds with the enzyme residues (Shie et al., 2005).

Another example of a dipeptide-based compound that can act as a protease inhibitor is dipeptidyl EP128533 (Zhang et al., 2006), which showed antiviral activity against SARS-CoV in Vero cells, with EC_50_ and CC_50_ values of 3.6 and >100 μM, respectively (Zhang et al., 2006). In accordance with that study, it was also demonstrated that EP128533 inhibited SARS-CoV with an EC_50_ of 1.4 μg mL^−1^ and CC_50_ > 100 μg mL^−1^ (Day et al., 2009). However, the compound was not efficient in reducing the effects of viral replication in BALB/c mice (Day et al., 2009). The authors proposed that EP128533 is relatively insoluble and that its lack of activity might be related to a low bioavailability in the animal models.

The dipeptides GC373 (dipeptidyl aldehyde) and GC376 (dipeptidyl bisulfite adduct salt from GC373) were also designed and synthesized as protease inhibitors of the 3CLpro enzyme (Kim et al., 2012). Their activity was assessed *in vitro*, and the results showed that GC373 inhibited HCoV-229E (EC_50_ of 0.2 μM), feline infectious peritonitis virus (FIPV, EC_50_ of 0.3 μM), MHV (EC_50_ of 2 μM), transmissible gastroenteritis virus (TGEV, EC_50_ of 0.3 μM), and bovine coronavirus (BCV, EC_50_ of 0.7 μM) (Kim et al., 2012). GC376 also inhibited HCoV-229E (EC_50_ of 0.15 μM), FIPV (EC_50_ of 0.2 μM), MHV (EC_50_ of 1.1 μM), TGEV (EC_50_ of 0.15 μM), and BCV (EC_50_ of 0.6 μM). The 3CLpro activity of these compounds against SARS-CoV was also analyzed. GC373 and GC376 inhibited enzymatic activity of SARS-CoV 3CLpro, with inhibition constants of 50% of 3.48 and 4.35 μM, respectively (Kim et al., 2012). However, the activity of these compounds was not evaluated using infected cells or animal models. Additionally, the effects of GC373 and GC376 were assessed against feline coronavirus WSU (FCoV-WSU) (EC_50_ values for GC373 and GC376 were 0.15 and 0.40 μM, respectively) (Kim et al., 2013). Moreover, the authors described that concomitant treatment with these compounds can improve the antiviral effect against feline coronaviruses and noted that, since the 3CLpro is conserved among CoVs, it might present broad-spectrum activity (Kim et al., 2013).

RNA-dependent RNA polymerase (RdRp) also figures as a promising target for antivirals. In viral replication, RdRp is responsible for catalyzing the replication of the viral RNA using a complementary RNA as a template. Therefore, compounds that interfere in this process are excellent drug candidates for treating viral infections (Ganeshpurkar et al., 2019). Nucleoside analogs of pyrimidine interfere in uridine triphosphate (UTP) metabolism, directly affecting viral replication (Murphy and Middleton, 2012), as demonstrated by β-D-N^4^-hydroxycytidine (NHC), which inhibited SARS-CoV (EC_50_ of 10 μM and CC_50_ > 100 μM) and HCoV-NL63 (EC_50_ of 400 nM and CC_50_ > 100 μM) (Barnard et al., 2004). NHC presented a potent antiviral activity against SARS-CoV-2 in infected Vero (IC_50_ of 0.3 μM and CC_50_ of > 10 μM) and Calu-3 cells (IC_50_ of 0.08 μM and CC_50_ > 100 μM) (Sheahan et al., 2020b). The authors assessed the broad-spectrum antiviral activity of NHC against MERS-CoV (IC_50_ 0.024 μM) and SARS-CoV (IC_50_ 0.14 μM) (Sheahan et al., 2020b) and also evaluated the NHC effect in SARS-CoV- and MERS-CoV-infected mice. NHC improved pulmonary function and decreased viral load in lung, and the authors proposed that NHC might be useful for emerging CoVs. Another pyrimidine analog with potential antiviral activity is 6-azauridine, which inhibited HCoV-NL63 replication in LLC-MK2 cells with an EC_50_ of 32 nM and CC_50_ of 80 μM (Pyrc et al., 2006).

Ribavirin is a synthetic nucleoside analog of guanosine used for the treatment of patients chronically infected by the hepatitis C virus (HCV) (PubChem, 2005c). The antiviral activities of ribavirin against several RNA viruses have been described, and it also presents broad-spectrum antiviral activities for CoVs (Chan et al., 2013; Shen et al., 2016). Its activities were described for SARS-CoV *in vitro* (EC_50_ of 20 μg mL^−1^ and CC_50_ > 200 μg mL^−1^) (Saijo et al., 2005). Nevertheless, no viral load reduction was observed *in vivo* when employing BALB/c mice (Barnard et al., 2006). The *in vitro* decrease of ribavirin efficacy was demonstrated to be associated with the excision of its nucleoside analogs by conserved coronavirus proofreading mechanisms (Ferron et al., 2017). Moreover, ribavirin showed good results for the treatment of critical MERS-CoV patients (Al-Tawfiq et al., 2014), and the combined treatment of ribavirin with type I Interferons (IFN-I) in primate models improved MERS disease symptoms (Falzarano et al., 2013b). Although ribavirin has been given as part of treatment regimens for SARS and MERS patients, meta-analyses of cases of study have found limited efficacy of its activities in treating patients with highly pathogenic coronavirus respiratory syndromes (Morra et al., 2018).

What is more, a nucleoside analog based on the acyclic sugar scaffold of acyclovir showed antiviral potential against coronaviruses (Tan et al., 2004). Peters and contributors demonstrated that this compound has powerful antiviral activity against MERS-CoV (EC_50_ and CC_50_ of 23 and 71 μM, respectively) and HCoV-NL63 (EC_50_ and CC_50_ of 8.8 and 120 μM, respectively) (Peters et al., 2015). However, the authors did not suggest mechanisms by which this analog impairs viral replication, leaving open to question whether it acts like its precursor acyclovir, impairing viral replication or by an alternative mechanism of action.

In terms of other drug options for the post-entry stages of the viral replicative cycle, it is possible to report the activities of Niclosamide, a drug used in antihelminthic treatment (Katz, 1977). Niclosamide presented antiviral activity on post-entry steps of SARS-CoV infection in Vero cells, with an EC_50_ of 1–3 μM and CC_50_ of 250 μM (Wu et al., 2004). Similarly, this compound suppressed the cytopathic effect of SARS-CoV at a concentration <1 μM and inhibited viral replication with an EC_50_ value of less than 0.1 μM in Vero E6 cells (Wen et al., 2007). Both authors suggested that Niclosamide impairs post-entry steps. However, this effect seems to not be related to an interaction with 3CLpro.

An additional potential compound is mycophenolic acid (MPA), an antibiotic derived from penicillium fungal species (PubChem, 2005b), which inhibited MERS-CoV replication in Vero cells with an EC_50_ of 2.87 μM (Hart et al., 2014). However, MPA was not active against SARS-CoV in either *in vitro* or *in vivo* assay (Barnard et al., 2006). The data suggested that MPA inhibits the enzyme IMP dehydrogenase, inducing apoptosis on alveolar macrophages and consequently inhibiting or suppressing cellular immune responses that are important for preventing or limiting viral infection (Barnard et al., 2006).

Bananins, on the other hand, are a class of adamantane-based compounds conjugated with a pyridoxal moiety (vitamin B6) (Kesel, 2003). These molecules showed effective inhibition of SARS-CoV in FRhK-4 cells, with EC_50_ < 10 μM and CC_50_ of 390 μM. On the basis of both time addition and ATPase assays, the authors proposed that the action of bananin is mainly on the post-entry step of virus replication and may be related to an effect on the helicase function and/or on components of cellular pathways (Tanner et al., 2005).

Finally, the nonstructural protein 10 (nsp10) of CoVs was described as being responsible for a stimulatory effect on nsp16, a classical S-adenosylmethionine-dependent (nucleoside-2’-*O*)-methyltransferase that acts in RNA binding or catalysis. The peptide TP29 was designed as a ligand to MHV nsp10 and presented broad-spectrum activity, inhibiting SARS-CoV (EC_50_ of 200 μM) and MHV (EC_50_ of 60 μM) replication in infected cell lines (Wang et al., 2015). The authors also assessed TP29 activity in MHV infected mice and demonstrated that treatment improved survival, decreased viral load in liver, and induced type 1 IFN. Based on these data, it was suggested that TP29 impaired nsp10/nsp16 2’-*O*-MTase activity, dysregulating the genome replication process.

### Looking Toward Host Machinery: A Different Approach to CoV Treatment

Targeting the host process during viral infection figures as a promising alternative for drug development and can play an important role in abrogating viral replication (Sayce et al., 2010; Ullah et al., 2019). Nitazoxanide is a broad-spectrum antiviral agent exploited for the treatment of, for instance, influenza A and B viruses, as well as Ebola virus (EBOV) (Rossignol, 2014; Jasenosky et al., 2019), with its activity related to the interference in host-regulated pathways during viral replication (Rossignol, 2016). *In vitro* studies demonstrated that Nitazoxanide was able to inhibit MERS-CoV in LLC-MK2 cells, with an EC_50_ of 0.92 μg mL^–1^. The authors suggested that nitazoxanide affects pro-inflammatory cytokines and suppresses their overproduction (Rossignol, 2016).

Another host-target compound is Saracatinib (AZD0530), a tyrosine kinase (SFK) inhibitor. This compound suppressed the early stages of the MERS-CoV replicative cycle in Huh7 cells (EC_50_ of 2.9 μM and CC_50_ > 50 μM), possibly by affecting the SFK pathways (Shin et al., 2018). SFK possesses a central function in signaling pathways such as ERK/MAPK and PI3K/AKT (Thomas and Brugge, 1997), which are strictly related to CoV infection. Therefore, SFK inhibition might promote viral clearance and can be used in association with other drugs (Shin et al., 2018).

Moreover, Cyclosporin A (CsA), a peptide with activity on the cyclophilin family of host enzymes (isomerases that act as chaperones) (PubChem, 2005a; Davis et al., 2010), inhibited SARS-CoV (100% inhibition at 16 μM), HCoV-229E (75% inhibition at 16 μM), and MHV (100% inhibition at 16 μM) in human and animal infected cell culture. CsA presented broad-spectrum antiviral activity against CoVs, and it seems to interfere with genome replication/transcription during CoV infections (de Wilde et al., 2011, 2013; Pfefferle et al., 2011). Alisporivir, a non-immunosuppressive cyclosporin A analog, inhibited the replication of SARS-CoV in Vero E6 infected cells at low-micromolar concentrations (EC_50_ of 8.3 μM; CC_50_ > 50 μM). This compound also showed broad-spectrum anti-CoV activity, inhibiting MERS-CoV EMC/2012 (EC_50_ of 3.6 μM), MERS-CoV N3/Jordan (EC_50_ of 3 μM), and SARS-CoV MA-15 (EC_50_ of 1.3 μM) *in vitro* (de Wilde et al., 2017). However, the authors demonstrated that Alisporivir did not enhance survival in CoV-infected mice (de Wilde et al., 2017).

Other biomolecules that are promising as drug antivirals are interference RNAs (iRNAs). These macromolecules are small non-coding RNAs associated with controlling the expression of genetic information (Wilson and Doudna, 2013) and have been described as promising candidates for the treatment of hepatitis B virus (HBV), HCV, HIV, and human T-cell lymphotropic virus (HTLV) infections (Ma et al., 2007; Shah and Schaffer, 2011; Sanan-Mishra et al., 2017). Short interference RNAs (siRNAs) were described as being effective for *in vitro* antiviral treatment of FIPV, a type of FCoV (McDonagh et al., 2011, 2015). Most recently, Li and colleagues designed and synthesized siRNAs that targeted the M and N genes of swine and porcine coronaviruses (SECoV and PDCoV, respectively). These siRNAs inhibited up to 99% of the expression of these proteins in both Vero and LLC-PK1 infected cells (Li et al., 2019). Additionally, synthetic siRNAs targeting the structural proteins E, M, and N of SARS-CoV have also been developed and showed reductions of the target gene expressions in Vero cells (Shi et al., 2005). Moreover, siRNAs targeting the structural proteins 7a, 7b, 3a, 3b, and S reduced SARS-CoV progeny in Vero cells by approximately 70% (Åkerström et al., 2007). The different authors propose that treatment with siRNAs can improve treatment-resistance among viruses and that these molecules can be designed to target multiple proteins, aiming at broad-spectrum activity.

### Ongoing Clinical Evaluations With Candidate Drugs Against SARS-CoV-2

The current situation of COVID-19 pandemic has accentuated the urgency of the demand for effective treatments. Based on previous data concerning activities against other viruses and empirical knowledge from treatments used in case reports, several drugs have entered clinical trial phases to access their therapeutic potential against SARS-CoV-2. In this section, we discuss the current knowledge on the most promising candidates for the treatment of COVID-19. Data for these drugs are summarized in Table 2.

The nucleoside analog Remdesivir (GS-5734) is a monophosphoramidate prodrug that has been described as having antiviral activity against the EBOV in non-human primates (Warren et al., 2016, 57). Its activity was assessed in human airway epithelial (HAE) cells infected with SARS-CoV (EC_50_ of 0.069 μM and CC_50_ > 10 μM) and MERS-CoV (EC_50_ of 0.074 μM and CC_50_ > 10 μM) and was demonstrated to inhibit RdRp of these viruses. Also, GS-5734 reduced infectious virus production of bat CoV by 1.5 to 2.0 log_10_ in HAE cells and reduced virus titers and virus-induced lung pathologies in a SARS-CoV assay *in vivo* (Sheahan et al., 2017). This compound also reduced the severity of MERS-CoV disease, virus replication, and damage in the lungs of rhesus macaques (De Wit et al., 2020). The clinical efficacy of GS-5734 has been assessed by several clinical trials in different countries like France (NCT04365725), Canada (NCT04330690), and the United States (NCT04292899), which have been conducted based on the first reported treatment of COVID-19 with Remdesivir in Washington, United States (Holshue et al., 2020). In the first findings from Wang and coworkers, which were from a randomized, double-blind, multicenter, and placebo-controlled trial with 255 patients, Remdesivir did not present significant antiviral effects against SARS-CoV-2, nor did it improve clinical outcomes (Wang et al., 2020d). To date, there are several active clinical trials registered in the PubMed database involving this compound. However, most of them presented no conclusive outcomes.

Another two candidates are Lopinavir and Ritonavir, which are protease inhibitors used in association to treat HIV infections (Cvetkovic and Goa, 2003; Mills et al., 2009). Lopinavir demonstrated antiviral activities, protecting cells from MERS-CoV infection (EC_50_ of 8 μM) and reducing viral loads in animal assays (de Wilde et al., 2014; Kim et al., 2015). Ritonavir also demonstrated anti-MERS-CoV activities with an EC_50_ of 24.9 μM (Sheahan et al., 2020a). It is important to point out that these results do not agree with another work that was unable to demonstrate *in vitro* antiviral activity of Lopinavir against MERS-CoV (Chan et al., 2013). In clinical assays for MERS-CoV, the association of Lopinavir with Ritonavir reduced adverse clinical outcomes and viral load in infected patients (Sheahan et al., 2020a; Yao et al., 2020a). In particular, for SARS-CoV, Lopinavir and Ritonavir presented a low to medium antiviral activity *in vitro*, and *in vivo* assays have not been performed yet (Yao et al., 2020a). In addition, Lopinavir and Ritonavir played an important role in the clinical outcome of SARS-CoV-infected patients by reducing symptoms and the period of hospitalization, representing a possibility for the treatment of SARS-CoV-2 (Chu et al., 2004). Cao and collaborators conducted a randomized clinical trial with 199 patients with severe COVID-19 (Cao et al., 2020b). Treatment of the patients with the association Lopinavir/Ritonavir did not improve symptoms, nor impaired detectable viral RNA when compared to standard care (supplemental oxygen, noninvasive and invasive ventilation, antibiotic agents, vasopressor support, renal-replacement therapy, and extracorporeal membrane oxygenation). Additionally, the treatment generated relevant adverse effects in some of the patients (Cao et al., 2020b). The authors proposed that the low efficacy of Lopinavir with Ritonavir might be associated with the time of administration, since individuals that were treated at the onset of the disease had improved clinical results (Cao et al., 2020b). Later, it was shown that the association of lopinavir and ritonavir with interferon-β1 and ribavirin to treat mild to moderate COVID-19 patients alleviated symptoms and decreased the durations of viral infection and hospital stay (Hung et al., 2020). This might be related to their inducing cellular immune response, impairing virus replication.

The type 1 interferons (IFN-I) have also been employed in clinical trials. These proteins belong to the cytokine family and are associated with the immune response in viral infections, thus playing major roles in antiviral immunity due to their immunomodulatory properties (Samuel, 2001). Therefore, they are commonly employed in the treatment of several diseases such as Hepatitis C (Kobayashi et al., 1993). There are two subtypes of IFN-I, alpha (IFN-α) and beta (IFN-β) (Samuel, 2001). IFN-β is associated with more potent activity (Chan et al., 2015) and is therefore capitalized on in the treatment for multiple sclerosis patients (Axtell et al., 2010). Due to its more potent inhibition profile, it was associated with potent antiviral effects against SARS-CoV, MERS-CoV, MHV, and HCoV-229E *in vitro* and *in vivo* (Sperber and Hayden, 1989; Vassão et al., 2000; Hensley et al., 2004; Falzarano et al., 2013a; Chan et al., 2015). IFN-β, in particular, has a protective effect in endothelial cells, up-regulating CD73 and consequently stimulating the anti-inflammatory molecules and maintenance of endothelial barrier (Bellingan et al., 2014; Sallard et al., 2020). However, a clinical trial with 301 patients showed that this effect was not sufficient to decrease mortality in SARS patients (Ranieri et al., 2020). Therefore, in SARS-CoV-2, IFN-β has been associated with other drugs in clinical trials, improving outcomes in COVID-19 patients as in lopinavir or ribavirin (Hung et al., 2020).

COVID-19 patients with mild to severe symptoms can develop hyperinflammation and hypercytokinaemia, which can lead to multiple organ failure and death (Mehta et al., 2020). The employment of corticosteroids has shown to be an alternative for overcoming the cytokine storm and hyperinflammation due to its activities on immune cells (Wilkinson et al., 1991). Such a capitalization was previously reported in SARS-CoV patients during the 2002–2003 epidemic (Chihrin and Loutfy, 2005). For SARS-CoV-2, corticosteroids can improve the clinical condition of patients, reducing hyperinflammation and the development of ARDS, with faster improvement of symptoms (Wang et al., 2020c; Zha et al., 2020). However, contrasting data concerning the efficacy of these drugs was described recently, showing that corticosteroids did not improve symptoms in COVID-19 patients (Zha et al., 2020). Moreover, dexamethasone emerged as a potential drug for treating COVID-19 patients, as shown by the results of a randomized, controlled, open-lab, and multicenter trial that assessed the effects of dexamethasone in 454 patients, described to date in pre-print findings (Horby et al., 2020). Data suggested that dexamethasone reduced death in one-third of patients in invasive mechanical ventilation and one-fifth of patients in non-invasive oxygen mechanical ventilation. However, it did not impair mortality in patients with no respiratory support (Horby et al., 2020). Other trials have been conducted, such as NCT043274011, but considering the preliminary results, the WHO suggested that treatment with dexamethasone may be applied during the third phase of COVID-19, when the hyperinflammation is determined, and respiratory support is needed.

Another antiviral drug assayed toward SARS-CoV-2 is Umifenovir, a licensed antiviral exploited for the prophylaxis and treatment of influenza viruses (Arbidol), which demonstrated good pharmacokinetics when absorbed by the organism (Proskurnina et al., 2020). This drug has an antiviral effect against SARS-CoV *in vitro* at 50 μg mL^–1^ (Khamitov et al., 2008). Lian and coworkers coordinated an observational study with 81 patients with moderate to severe SARS-CoV-2 infection (Lian et al., 2020) that demonstrated that Umifenovir neither shortened the hospitalization period nor improved prognosis in infected patients (Lian et al., 2020).

Broad-spectrum drugs used against parasitic infections such as Ivermectin (Campbell, 2012; Laing et al., 2017) have also been investigated due to their antiviral activity against Dengue virus (DENV), Influenza A viruses, Chikungunya virus (CHIKV), and HIV (Tay et al., 2013; Götz et al., 2016; Varghese et al., 2016; Caly et al., 2020). The activity of Ivermectin is based on impairing several stages of viral replication, for instance, interfering with nonstructural proteins (Varghese et al., 2016). Caly and collaborators assessed the effect of Ivermectin on SARS-CoV-2 replication in Vero cells, showing that, at 5 μM, the compound presented no toxicity to cells and inhibited up to 99% of viral replication by a possible antiviral effect on viral release, which is consistent with previous data on its activity against other RNA viruses (Tay et al., 2013; Caly et al., 2020). Clinical trials have been conducted in different medical centers in Argentina (NCT04381884), Mexico (NCT04391127), Spain (NCT04390022), and the United States (NCT04374279) to assess the clinical implications of the use of Ivermectin for COVID-19. However, to the best of our knowledge, there are no published results on this topic. NCT04343092, a phase 1 clinical trial in Iraq, was conducted to its completion and evaluated the efficacy of Ivermectin in COVID-19 patients, so the results might be published soon.

According to Guan and colleagues, approximately 15.7% of Chinese patients with COVID-19 developed severe pneumonia and cytokine release syndrome (CRS), an important factor leading to rapid progression of the disease (Chousterman et al., 2017; Guan et al., 2020). In this context, one of the key cytokines involved in infection-induced cytokine storm is interleukin 6 (IL-6) (Scheller and Rose-John, 2006; Zhang et al., 2020a). Tocilizumab is an IL-6 receptor antagonist approved by the US FDA for the treatment of severe CRS (Grupp et al., 2013) and figures as an interesting drug to treat the cytokine storm caused by SARS-CoV-2 (Zhang et al., 2020b). The treatment of patients with severe COVID-19 with Tocilizumab presented no complications in the 21 assisted patients, with an average age of 56.8 ± 16.5 and no history of illness deterioration or death. Thus, it immediately improved the clinical outcome and appeared to be an effective treatment for reducing mortality (Xu et al., 2020). Another study employing the treatment of COVID-19 patients with Tocilizumab for 14 days reinforced these observations. The treatment was observed to cause an effective decrease in inflammatory markers, radiological improvement, and a reduction in ventilatory support requirements for these patients (Alattar et al., 2020). Additionally, Toniati and collaborators administered Tocilizumab in 100 patients in Italy (average age of 62 years old) who had been diagnosed with COVID-19 pneumonia and ARDS and required ventilatory support. Overall, at 10 days of follow-up, the respiratory condition was improved or stabilized in 77% of the patients, and, based on these data, the response to this drug in patients with severe COVID-19 was rapid, sustained, and associated with significant clinical improvement (Toniati et al., 2020).

Chloroquine is a 9-aminoquinole that increases the pH in acidic vesicles (Mauthe et al., 2018) and possesses antiviral activities against HIV and other viruses (Jacobson et al., 2016; Al-Bari, 2017). Chloroquine was described as an entry inhibitor of SARS-CoV infection in Vero cells and prevented cell-to-cell spread of the virus (Vincent et al., 2005). Furthermore, it affected the entry and post-entry stages of the replicative cycle of FCoV in *Felis catus* cells and monocytes. Additionally, an *in vivo* study in cats demonstrated that treatment with chloroquine improved the clinical score of treated groups when compared to the untreated group (Takano et al., 2013). Chloroquine also had its anti-CoV activities tested in Vero cells (EC_50_ of 5.47 μM) (Wang et al., 2020b; Yao et al., 2020b). Despite the performance of chloroquine *in vitro*, clinical studies conducted in China and France showed contradictory clinical data (Chen J. et al., 2020; Chen Z. et al., 2020; Gao et al., 2020; Molina et al., 2020). Gao and collaborators indicated that chloroquine phosphate was recommended to treat COVID-19-associated pneumonia only during urgent clinical demand because of its antiviral and anti-inflammatory activities (Gao et al., 2020). Hydroxychloroquine is an analog of chloroquine that was described as having antiviral activity, inhibiting SARS-CoV-2 *in vitro* with an EC_50_ of 0.72 μM (Liu et al., 2020; Yao et al., 2020b). In clinical trials, an open-label non-randomized study by Gautret and colleagues affirmed that hydroxychloroquine reduced symptoms from SARS-CoV-2 patients and that association with azithromycin could reinforce its effects (Gautret et al., 2020). However, these results have been questioned. The study had a small sample size, and there were limitations in the methodologies (Juurlink, 2020).

Recent studies have been contradicting the safety of chloroquine and hydroxychloroquine use, as these drugs presented severe side effects that interfered with their clinical use, even during short-course therapies (Juurlink, 2020; Liu et al., 2020). Apart from the mild adverse effects, such as pruritus, nausea, and headache, these drugs can predispose patients to life-threatening arrhythmias, an effect that may be enhanced by concomitant use of azithromycin (Chorin et al., 2020). Both chloroquine and hydroxychloroquine interfere with ventricular repolarization, leading to prolongation of the cardiac QT interval and an increased risk of torsades de pointes (TdP), which is a risk especially for patients with cardiac disease, for children, or for those taking other drugs that delay repolarization (Mzayek et al., 2007; Pukrittayakamee et al., 2014; Juurlink, 2020; Ursing et al., 2020). Others possible types of damage are hypoglycemia, even in non-diabetic patients (Unübol et al., 2011; El-Solia et al., 2018); neuropsychiatric effects, including agitation, insomnia, confusion, paranoia, depression, psychosis, and suicidal ideation (Mohan et al., 1981); hypersensitivity reactions, such as severe cutaneous adverse reactions (Cameron et al., 2014; Girijala et al., 2019); and drug–drug interactions, which are improved by genetic variability (genetic polymorphisms of hepatic cytochrome P450 enzyme 2D6 (CYP2D6), responsible for chloroquine metabolization) (Kirchheiner et al., 2008; Lee et al., 2016). There is a lack of reliable information on target concentrations or doses for COVID-19, and so doses that proved effective and safe in malaria for both adults and children are considered for the treatment (Smith, 2020). Recently, the WHO stopped the hydroxychloroquine arm of the Solidarity trial to treat COVID-19 based on an absence of effectiveness in reducing the mortality of hospitalized COVID-19 patients (WHO, 2020c). Besides, the FDA also cautioned against the administration of hydroxychloroquine or chloroquine in COVID-19 patients, mainly due to the risk of heart rhythm issues (FDA, 2020). From these results, it is evident that the use of these drugs for COVID-19 requires further investigation.

An alternative treatment for COVID-19 is the utilization of convalescent plasma (CP) (Chen L. et al., 2020). This treatment refers to plasma therapy based on plasma or plasma derivatives, obtained from donors who were previously infected and have developed antibodies. This plasma/derivative is, in its turn, transfused into individuals with acute SARS-CoV-2 infection (Garraud, 2017; Cao and Shi, 2020). Even though the mechanism of action of convalescent plasma therapy is not fully understood, it presented great results in the treatment of patients with SARS during the SARS-CoV outbreak in Hong Kong in the early 2000s (Cheng et al., 2005). It is possible that the efficacy of CP therapy is due to the fact that the antibodies from convalescent plasma might suppress viremia (Chen L. et al., 2020). Duan and colleagues reported CP transfusion to rescue ten severe cases of SARS-CoV-2 adult patients. The study showed that one dose (200 mL) of CP significantly increased or maintained the neutralizing antibodies at a high level, leading to the disappearance of viremia in 7 days. Clinical symptoms rapidly improved within 3 days, and radiological examination showed varying degrees of absorption of lung lesions within 7 days. According to these results, CP can also provide a promising rescue option for severe COVID-19 (Duan et al., 2020). However, the author suggested key points to guarantee the effectiveness of CP therapy: Ab titers and the treatment time point. Firstly, taking into consideration previous knowledge from MERS-CoV CP therapy, Abs in plasma donor must have a titer equal or higher of 1:80 (Ko et al., 2018). This titer is only found in recently recovered patients, since antibody levels decrease 4 months after the disease. Secondly, patients receiving CP treatment prior to 14 days post-infection responded better than patients treated after 14 days (Duan et al., 2020).

## Perspectives

This review aimed to summarize and discuss data from the literature regarding compounds that possess anti-CoVs activities and that could be further exploited for the treatment of human and animal CoVs. Furthermore, we described ongoing clinical trials for SARS-CoV-2 in order to elucidate the current findings and discussed the relevant features concerning candidate drugs against SARS-CoV-2.

As previously mentioned, most human-related CoVs emerged by zoonotic transmission from animals (Huynh et al., 2012; Coleman and Frieman, 2014; Reusken et al., 2016). Since *Coronaviridae* seem to have a very well conserved genome and structures among their viruses (Huentelman et al., 2004a; Guan et al., 2012; Yang and Leibowitz, 2015; Madhugiri et al., 2018), it is possible to hypothesize that compounds with antiviral activities against different human and/or animal CoVs (broad-spectrum activity) could be potential candidates for SARS-CoV-2 treatment. In a less optimistic scenario, the chemical structures of such compounds and their pharmacological outcomes have the potential to set some light on the drug design of possible anti-SARS-CoV-2 drugs.

Among the strategies for drug design, targeting host-immune factors or using iRNAs figure as promising alternatives for antiviral drug development. Also, the exploitation of *in silico* studies for drug screening to seek specific targets, as well as for a better comprehension of their interactions with viral biomolecules, has been shown as a promising tool for expediting drug development. By narrowing down the number of drug candidates, *in silico* studies have the potential to avoid the laborious and generally costly synthesis of many of these compounds (Lengauer and Sing, 2006; Villegas-Rosales et al., 2012). Nevertheless, several predicted compounds in the literature have only been screened by *in silico* and/or interaction assays (Chen et al., 2005; Kaeppler et al., 2005; Lee et al., 2005; Kim et al., 2012; Arya et al., 2020; Balasubramaniam and Reis, 2020), which ultimately hinders the proper assessment of the antiviral activities of the compounds. Therefore, it is imperative that these studies be associated with *in vitro* and *in vivo* assays in order to confirm the predicted activities in biological models and also to evaluate pharmacological outcomes (National Research Council (US) Committee on Applications of Toxicogenomic Technologies to Predictive Toxicology, 2007). Therefore, this review encompassed only compounds that have been evaluated by, at least, *in vitro* models (Table 1).

In this context, from the molecules and drugs described as having *in vitro* activity, we highlighted the most promising to suggest further evaluation using *in vivo* systems of CoV infection, especially SARS-CoV-2 infection. The compounds are: NAAE, Glycyrrhizin, 2-acetamido-α-D-Glucopyranosylamine derivative, Tetrahydroquinoline oxocarbazate (CID 23631927), SSAA09E1, 2 and 3, Emodin, Eremomycin 27 and 29, Mucroporin-M1, Monoclonal antibody 47D11, AVLQSGFR, Phe-Phe dipeptide inhibitor C (JMF1521), GC373 and 376, 6-azauridine, Acyclic sugar scaffold of acyclovir, and Bananins. As described above, these compounds were capable of significantly impairing CoV infection in cell cultures and might enable important progress into the treatment of described CoVs as well as viruses that might be responsible for future viral outbreaks.

Here, we also described compounds that were evaluated *in vivo* to elucidate their role in the pathogenesis of CoVs as well as to assess possible adverse effects. It is important to emphasize that there is a lack of *in vivo* model assays, representing a delay in anti-CoV drug development, which directly impacts the SARS-CoV-2 pandemic. Here, we identified some studies that employed animal models, such as in Balb/c mice and C57BL/6, to evaluate the antiviral effect of compounds in CoV infection (Cinatl et al., 2003; Saijo et al., 2005; Barnard et al., 2006; Zhang et al., 2006; Day et al., 2009; Hart et al., 2014). The *in vivo* assays allow the gathering of knowledge regarding the ADMeTox profile of these compounds in complex biological systems, the viral titers in different organs, host immune responses to the infection, and also potential tissue damage caused by the viruses in the presence or absence of candidate drugs, which represents an advance in understanding pathologies caused by viral infections (Adachi and Miura, 2014). It is also important to emphasize that protocols used in studies of animal-related viruses are not easily translated onto human CoVs, since these viruses are classified to different biological safety levels, representing a risk of infection to scientists (Bayot and King, 2020; CDC, 2020b). Additionally, the pathologies induced by animal CoVs are mostly related to gastrointestinal symptoms, differently to what is observed for human-related CoVs, which mostly affect the upper respiratory system (Pedersen et al., 1984; Coleman and Frieman, 2014). The development of refined and secure protocols to study SARS-CoV-2 infection and its treatment options is required. Bearing in mind the obstacles cited above, assessment of the effect in animal models and further translation to humans remains one of the main challenges.

However, some of the studies were able to assess the antiviral effects of some compounds *in vivo*. The most relevant compounds we propose that may represent immediate candidates to clinical trials, considering the urgency of COVID-19, are Griffithsin (GRFT), β-D-N^4^-hydroxycytidine (NHC), TP29, Cyclosporin A (CsA), Alisporivir, iRNAs, Saracatinib, Tizoxanide, Nitazoxanide, Niclosamide, and Ribavirin. These compounds abrogated CoV infection *in vitro* and *in vivo* and improved the symptoms and survival of animals. In addition, Saracatinib, Tizoxanide, Nitazoxanide, Niclosamide, and Ribavirin are molecules licensed to treat diseases such as those from viral and helminthic infections or Alzheimer’s disease, representing possibilities for clinical trials as repurposed drugs.

Regarding clinical trials, most drugs discussed in this review presented adverse effects such as nausea, headache, diarrhea, urticaria, pathologies related to the gastrointestinal system, and interference with liver enzymes (Ruiz-Irastorza et al., 2010; Takano et al., 2013; Roques et al., 2018; Yao et al., 2020a). Remdesivir, Lopinavir and Ritonavir, and Umifenovir are drugs employed for the treatment of other viral infections such as EBOV and SARS-CoV, but, in the clinical trials with COVID-19 patients, these treatments did not reduce symptoms and/or decrease viral load. Tocilizumab, Chloroquine, and Hydroxychloroquine have been demonstrated to inhibit SARS-CoV-2 *in vitro* and, in some clinical trials, reduced COVID-19 symptoms, the period of hospitalization, and the viral load in patients despite the strong adverse effects of Chloroquine (Table 2). Even so, recent studies are contradicting the safety profiles of Chloroquine and Hydroxychloroquine, since they might cause arrhythmia in patients, representing risk for a considerable number of patients (Juurlink, 2020).

Ongoing studies have been evaluating IFN-β and Ivermectin as treatments against COVID-19. IFN- β can be associated with other drugs, collaborating to control immune response against the viral infection (Table 2). On the other hand, corticosteroids, such as dexamethasone, sound promising, but there are some issues related to their use. These compounds induce immunosuppression and, when administered during initial phases (viral replication), might dysregulate T-cell production and activation of B cells for antibody secretion, which are essential for viral clearance (Cohn, 1991; Giles et al., 2018). Furthermore, convalescent plasma therapy is an alternative approach that presented positive effects in studies on SARS-CoV-2/COVID-19 patients. However, its safety is not well defined due to donor-dependent variability and compatibility (antibody titers and other factors vary among donors), which might cause severe adverse effects in lung and cardiovascular system and, in some cases, may even transmit diseases (Roback and Guarner, 2020).

Despite the finding regarding these drugs, it is important to take some aspects into consideration: i) the trials were generally conducted with a significant number of patients in each study, but potentially not enough to expand the results to public healthcare; ii) some of the studies were observational, which means they were based on public data that may not be well documented, leaving information gaps about particular health issues; additionally, the outcomes in patients are defined by their own circumstances, and not by an investigator; iii) some studies were not placebo-controlled and double-blind, so the placebo effect cannot be discarded (Kernan et al., 1999; Hess and Abd-Elsayed, 2019); iv) the trials were conducted by selecting a group of COVID-19 patients, considering mild, moderate or severe cases, and different outcomes can be expected in each situation since viral load, the progression of the disease, and immune response are additional factors (Kernan et al., 1999; Hess and Abd-Elsayed, 2019). Therefore, drugs with no effect in severe cases cannot be rejected as a possible treatment in mild to severe cases. When these aspects are not considered, the investigators might be open to commiting type I or II error in trials (Kernan et al., 1999; Hess and Abd-Elsayed, 2019). For that matter, it is also important to consider that SARS-CoV-2 is a new virus and that we currently have limited knowledge about its physiopathology. Finally, the development of new treatment options is critical, and efforts have been focused on targeting therapies that aim to improve patient outcome by increasing antiviral activity associated with minimal toxicity.

Another point to be considered in CoV treatment is that RNA viruses are known to have high levels of mutations (error rate) in the replication process (Ganeshpurkar et al., 2019). This can result in resistance to antiviral treatment, as observed for HIV, HCV, and Influenza viruses (Laplante and St George, 2014; Li and Chung, 2019; Olearo et al., 2019; Takashita, 2020). A recent study in pre-print pointed to the genomic variability of SARS-CoV-2 and the intra-patient capacity of polymorphic quasispecies, which may offer resistance to antiviral drugs (Karamitros et al., 2020). In addition, previous studies demonstrated that the use of Chloroquine analogs for decades against malaria has established chloroquine-resistant Plasmodium strains (Stocks et al., 2002; Al-Bari, 2017; Aguiar et al., 2018). Due to the beneficial immunomodulatory effects of analogs on the severe inflammatory complications of several viral diseases, such as HIV and SARS-CoV infections, these drugs have been tested indiscriminately (Jacobson et al., 2016; Al-Bari, 2017). However, there is a possibility that prophylactic exposure to pro-apoptotic chloroquine drugs caused natural selection for strains of viruses and other parasites that have enhanced anti-apoptotic abilities (Parris, 2004). Despite the side effects, the wide use of some drugs during the SARS-CoV-2 pandemic might raise concerns regarding the emergence of resistant viral strains in the future, and we emphasize the lack of information on the resistance associated with these drugs in the treatment of viral infections.

## Conclusion

The spread of SARS-CoV-2 worldwide is classified as a pandemic and represents a threat to global public health. By July 4, 2020, SARS-CoV-2 had infected 10,922,324 people and had caused 523,011 deaths around the world (WHO, 2020b). In this context, compounds described to possess antiviral activity against human and/or animal coronaviruses could provide relevant information for the development of novel SARS-CoV-2 treatments. Herein, we presented and discussed the most promising compounds that can figure as possible candidates for clinical trials. Moreover, ongoing clinical trials evaluating possible COVID-19 therapies were also highlighted.

From what was presented in this review, a plethora of different potential compounds can be capitalized as possible drugs or even set points for further drug development seeking to mitigate the SARS-CoV-2/COVID-19 outbreak. However, time, resources, and new experimental protocols are essential for advancing an efficacious treatment. In addition, and despite the urgency of treatment protocols, it is important to point out the striking need for the establishment of fail-proof regulatory initiatives that could prevent impacts on the healthcare of patients that could, otherwise, be avoided by a more stringent control.

In this context, this review describes drugs that might be overlooked for future analysis and could possibly become effective antiviral treatments. As a final remark, we conclude that, to date, there is no “one hundred percent” effective antiviral therapy against SARS-CoV-2/COVID-19 and that further research is needed to achieve the best therapeutic protocol, which may not be based on a unique drug but rather on a combination of active antivirals.

## Author Contributions

IS: drafting the manuscript and literature review. VG: drafting the manuscript and illustration. FB, RS-S, and AJ: critical revision, editing, and approval of the final version. All of the authors read and approved the final manuscript.

## Conflict of Interest

The authors declare that the research was conducted in the absence of any commercial or financial relationships that could be construed as a potential conflict of interest.
